# What is known so far about bull sperm protamination: a review

**DOI:** 10.1590/1984-3143-AR2021-0109

**Published:** 2022-11-04

**Authors:** Carlos Alonso Paco Nagaki, Thais Rose dos Santos Hamilton, Mayra Elena Ortiz D Ávila Assumpção

**Affiliations:** 1 Departamento de Reprodução Animal, Faculdade de Medicina Veterinária e Zootecnia, Universidade de São Paulo, São Paulo, SP, Brasil

**Keywords:** protamine, spermatozoa, DNA fragmentation, spermatogenesis, bull fertility

## Abstract

Sperm routinary fitness evaluation is not sufficient to predict bull reproductive capacity as they present differences in fertility up to 40%. Among the defects which compromise spermatozoa functionality, new approaches consider the study of sperm chromatin, which is the core structure containing paternal genetic information. Sperm chromatin needs to be compacted to maintain the integrity of DNA, which occurs by binding nucleoproteins with high affinity to DNA. In the last stages of sperm maturation, chromatin is hyper-compacted by basic proteins called protamines in a process named protamination. In this review, we summarized intrinsic and extrinsic factors that are suggested to influence protamination in bull spermatozoa, considering old and new evidence from human and murine spermatozoa. Also, the current approaches to evaluate bull protamination and its relationship with fertility were described. Nevertheless, the physiological mechanisms of protamination are still poorly understood.

## Introduction

The use of assisted reproduction techniques (ARTs) in cattle production is expanding as it promotes genetic gains by breeding dams with sires of high genetic potential (Widayati, 2012). However, the outcome of ARTs is unpredictable as these techniques are highly susceptible to undetected alterations in female and male gametes (Seegers et al., 1994; Inchaisri et al., 2010; Daly et al., 2020).

Spermatozoa is a complex cell that could have hidden alterations that can delay genetic improvement since AI centers commercialize straws from selected bulls for multiple inseminations. This arises a big concern inside these centers, as normozoospermic bulls show up to 25% of fertility differences when tested *in vivo* (Kidder et al., 1954; Larson and Miller, 2000; Alahmar, 2019).

As each ejaculate shows a heterogenous cell population, identifying and characterizing the defects become a key step to assess spermatozoa viability. There are defects named as compensable because it is possible to achieve minimum pregnancy rates by increasing cell concentration in inseminating doses (Saacke, 2008). Examples of these are morphological and structural alterations that impair spermatozoa's movement through the female reproductive tract and are also suspected of blocking capacitation and spermatozoa-egg recognition (Freundl et al., 1988; Saacke, 2008). In this same ejaculation are present other defects considered non-compensable because their negative impact can’t be compensated by increasing cell concentration (Kastelic, 2013). This classification considers defects in chromatin compaction and plasma membrane structures because they can impair fertilization and block early embryogenesis (DeJarnette, 2005; Flowers, 2013), and sperm chromatin alterations in bull spermatozoa, as it influences negatively the reproductive outcome causing decreased cleavage rates and delayed pronucleus formation (Shamsuddin and Larsson, 1993; Eid et al., 1994). It is essential to maintain the integrity of sperm chromatin as it contains paternal DNA and early signaling for embryo development, then DNA breaks or DNA fragmentation makes unfeasible fertilization and compromises fertility (Oleszczuk et al., 2013; Choi et al., 2017). The causes of DNA susceptibility and failures in chromatin compaction are suggested to be a consequence of impaired nuclear remodeling due to failures in histone replacement by protamines in the final phase of spermatogenesis (Gosálvez Berenguer et al., 2008).

Among human and murine spermatozoa, the most studied models, failures in nuclear remodeling result in defective cells with increased susceptibility to sperm DNA fragmentation (Björndahl and Kvist, 2010). Despite this, spermatozoa still have the fertilizing capacity, but if the severity of sperm DNA damage exceeds the oocyte’s repair capacity, blockage of early embryonic development may occur (Fatehi et al., 2006).

The objective of this review was to describe the current approaches to bull protamination evaluation and its relationship with fertility.

### Remodeling of sperm DNA

**Figure 1:**
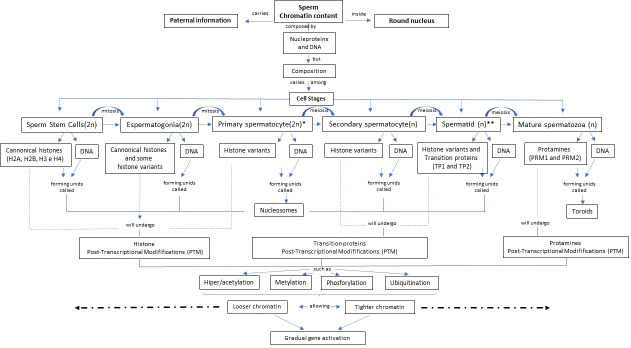
Concept map of proposed sperm chromatin dynamics and content along with spermatogenesis phases in bovine spermatozoa. *Primary spermatocyte: This stage considers leptotene, zygotene, pachytene, and diplotene before the two consecutive meiotic phases. **Spermatid: This stage considers round and elongated spermatid preceding the protamine deposition.

Preservation of male genetic information contained in chromatin is achieved by DNA binding to various nucleoproteins (histones, transition proteins, and protamines) throughout sperm maturation (Rathke et al., 2014). In the early stages of spermatogenesis, first sperm stem cells (SSC) located close to the seminiferous tubules will differentiate into early spermatogonia (2n), whose DNA is coiled into octamers of canonical histones (H2A, H2B, H3, and H4) resulting in units called nucleosomes (Talbert and Henikoff, 2010). As spermatogenesis progresses (Figure 1), canonical histones will undergo post-transcriptional modifications (PTM) that will destabilize those nucleosomes, allowing relaxation of chromatin structure and promoting replacement with histone variants and testis-specific (Bao and Bedford, 2016). After the meiotic phase, late spermatocytes will produce haploid round spermatids with transient-remodeled nucleus chromatin which suffered histone substitution for transition proteins 1 and 2 (TNPs) until elongated spermatid (n) stage (Meistrich et al., 2003).

As shown in Figure 1, elongated spermatids in late spermiogenesis will undergo morphological changes to compact the nuclear content and maintain the hydrodynamic and spermatozoa characteristic shape. To this end, TNP1 and TNP2 will also suffer post-transcriptional modifications as hyperacetylation that facilitates the replacement by basic and high-affinity proteins called protamines (PRMs) to the DNA (Balhorn, 2007). PRMs are low molecular weight nucleoproteins composed of arginine and oxidized cysteine, which are tightly bound to each other due to disulfide bridges on the cysteine residues. Also, their composition results in a highly positively charged protein with a high affinity for DNA (Balhorn et al., 2000).

The protamination process involves the species-specific participation of two protamine families: protamine 1 (PRM1) and protamine 2 (PRM2, PRM3, and PRM4). The PRM1 is usually synthesized as a mature protein. PRM1 is composed of 50 amino acids with 3 main domains: a central arginine-rich domain; a high affinity for DNA, flanked by serine and cysteine residues domain; and the last containing threonine segments and several phosphorylation sites (Steger and Balhorn, 2018). Also, PRM1 is found in the sperm nucleus of almost all mammals and has a highly conserved structure between species. On the other hand, PRM2 usually is synthesized as a precursor and it is present in spermatozoa of a few species such as mice, equine, primates, and humans (Steger and Balhorn, 2018). In bovines, spermatozoa protamination was attributed entirely to PRM1, until recent reports of PRM2 translation in both testicular and sperm tissue (Hamilton et al., 2019).

Protamine substitution in mature bull spermatozoa would establish a strong and protected structure, believed to be a result of an evolutive mechanism due to high selection pressure. For this reason, it is important to measure protamination, as it would be a checkpoint to assure bull fertility. Immature spermatozoa inside the testis or in epididymal transit still show low protamine binding (CMA3 assay) which correlates with high rates of DNA susceptibility to fragmentation (Fortes et al., 2014).

Protamines have an important role in maintaining male fertility, as this hyper-compaction would preserve the integrity of sperm chromatin throughout the female tract until reaching the oocyte (Agarwal et al., 2003). At the same time, recently, human and bull protamines are being used as sperm biomarkers to assess fertility considering the differences in the amount of PRM1 and PRM2 in sperm chromatin (Fraga et al., 1996; Pardede et al., 2020).

### Chromatin content

Chromatin compaction is a late-spermatogenesis process with species-specific signaling, so the conformation and amount of each protamine in sperm chromatin will differ between species (Oliva, 2006). Mammal spermatozoa can show up to 40% of structural differences among protamine structures and unique PRM1:PRM2 ratios for each specie (Steger and Balhorn, 2018). Protamines undergo structural modifications after translation, where cysteine residues are oxidized producing inter and intra-protamine disulfide bonds (Vilfan et al., 2004). This variability related to the number of cysteine residues between species could result in a greater or lesser degree of chromatin compaction (Bennetts and Aitken, 2005; Villani et al., 2010).

In spermatozoa of some species like humans, the physiological replacement of protamines in spermatozoa is incomplete, generating a fraction of DNA (≅ 15%) still attached to nucleosomes (Hammoud et al., 2009; Erkek et al., 2013). The mechanisms involved in histone retention are still poorly elucidated, but it is believed that some histones with post-transcriptional modifications (PTM) can evade replacement by the action of transcription factors such as BORIS (Brother of the Regulator of Imprinted Sites) and CTCF (CCCTC-binding factor) while generating a form of epigenetic inheritance (Pugacheva et al., 2015). In addition to this, the presence of promoters and genes required for early embryonic activation has been reported to be further linked to nucleosomes with PTM-histones (H3K4me3, H3K4me2, and H3K27me3) in human and bovine spermatozoa (Samans et al., 2014; Jung et al., 2017). Another important histone-variant along spermatogenesis is the T2HB, which is suggested to be present in the whole spermatogenesis, as it would facilitate nucleoproteins substitutions and chromatin remodeling in mouse and bovine spermatozoa (Shinagawa et al., 2015; Kutchy et al., 2017).

Sperm chromatin content is considered a unique mosaic for each species and even for breeds as the proportion of nucleoproteins varies among individuals, thanks to the presence or absence of regulatory transcription factors (Oliva et al., 2009). These particularities are even more important as chromatin composition could vary between individuals with different fertility scores. To acknowledge this, several protein-related studies were compiled, and it was observed quantitative differences between proteins related to acrosomal function, capacitation processes, seminal plasma, and protamine content among bulls used in artificial insemination programs with fertility problems (Harayama et al., 2017). As these recent approaches describe the importance of sperm chromatin integrity in bulls, the next step would be identifying sperm chromatin molecules or integrity analyses to verify chromatin status or using them as biomarkers in bull spermatozoa. Currently, biomarkers in chromatin are used to identify changes in somatic cells of human tissues for diagnosing, preventing, and prognosticating, and even as a therapeutic approach for human diseases (Hlady and Robertson, 2016).

### PRM1:PRM2

It is important to acknowledge that mammals have more protamines (85%-99%) bound to DNA molecules when compared to humans, which have more DNA still attached to histones (Oliva, 2006). Among the proteins present in the nucleus, human sperm protamines are considered an important biomarker, as retrospective studies show the ideal ratio of PRM1: PRM2 close to 1 correlated to fertile individuals. Also, the same study reported alterations in the PRM1:PRM2 ratio and higher rates of DNA fragmentation in men with infertility/subfertility antecedents, which decreased the success of *in vitro* reproduction techniques, such as intracytoplasmic sperm injection (ICSI) and in vitro fertilization (IVF) (Balhorn et al., 1988; Evenson and Wixon, 2006). Afterward, it was described that PRM1:PRM2 is correlated with other analyses (CMA3 and DFI) that evaluate DNA integrity, so it is suggested to be a strong predictor of men's fertility (Amor et al., 2018).

Moreover, experiments using murine spermatozoa as a biological model also observed a complementary role of the binomial PRM1:PRM2 (Cho et al., 2003). These studies with murine spermatozoa described that heterozygous animals for PRM1+/- and homozygous for PRM2-/- were infertile with high DNA fragmentation rates as presenting spermatozoa with decreased motility (Schneider et al., 2016; Takeda et al., 2016). Contrary to human sperm, PRM2 expression in murine is predominant and influences the ideal ratio (PRM1:PRM2), resulting in a value close to 0.6 (Arévalo et al., 2021).

In bulls, chromatin compaction was attributed only to PRM1, while PRM2 was believed to be nonfunctional (Maier et al., 1990). Recently, gene and protein expression of PRM2 has been described in bovine spermatozoa, even at low amounts (Hamilton et al., 2019). Despite this, PRM2 function is hypothesized to let loose some DNA regions where some genes would need rapid transcription before and mainly after fertilization and would be the target of epigenetic markers along sperm chromatin compaction (Hamilton et al., 2019). As PRM1 is the predominant nucleoprotein in bull spermatozoa, it would play a key role in fertility as decreased gene and protein expression is related to bulls with lower conception rates or lower reproductive success, as already described for human and murine species (Aoki et al., 2006; Bissonnette et al., 2009; Feugang et al., 2010). In addition to this, Dogan et al. (2015) reported a positive correlation between protamination levels with the fertility of bulls used in AI programs, as well as an aberrant distribution of PRM1 in spermatozoa with morphological defects.

Therefore, some studies mention the relationship of deleterious morphological defects with failures in protamination or higher rates of DNA fragmentation in bovine spermatozoa (Enciso et al., 2011; Carreira et al., 2015). Also, it has been reported a protamination difference (using Chromomycin A3 assay) among bulls of different fertility, although being still low values (Castro et al., 2018). Despite the complementary role of PRM1 and PRM2 in bull spermatozoa are still unclear, the lack of protamination which is evaluated by the CMA3 test is an important component in current models of fertility prediction (Llavanera et al., 2021).

### Inefficient protamination

Spermatozoa protamination in mammals occurs at the last epididymal passage and is part of the last structural modifications of the spermatozoa to acquire the hydrodynamic characteristics that allow it to fertilize the oocyte (Dadoune, 2003). However, the regulatory mechanisms involved in nucleoprotein substitution are poorly elucidated. Despite the lack of knowledge, it is known that there are intrinsic and extrinsic factors involving spermatogenesis that can alter sperm compaction and protamination (summarized in Table 1).

**Table 1:** Summary of references about impaired protamination.

**Nature**	**Causes of defective protamination**	**Specific pathway**	**Reference**
Intrinsic	Irregular post-transcriptional modifications (PTM) in canonical histones	FSH and testosterone synergy	Xing et al. (2003)
		H3T	Yuen et al. (2014)
		TH2B	Ausio et al. (2016)
	Alterations in the gene sequence of PRM1 and PRM2	VLRs of altered length	Hammoud et al. (2007)
		SNPs	Ravel et al. (2007)
	Alterations in protamination regulatory pathways	Via CREM	Blendy et al. (1996)
		Via MSY2	Yu et al. (2002)
		Via PRBP	Lee et al. (1996)
Extrinsic	Oxidative Stress	High temperature	Hamilton et al. (2018); Rahman et al. (2011)
		Exposure to oxidizing agents	Wu et al. (2020)
		Exposure to chemotherapeutic agents	Codrington et al. (2007)
		Dietary vitamin effect	Gao et al. (2021)

### Intrinsic factors

Throughout spermatogenesis, canonical histones undergo post-transcriptional modifications (PTM) within the nuclear structure (Lewis et al., 2003; Rathke et al., 2014). These PTM along with testis-specific histones regulating genes are required for spermatogenesis and chromatin remodeling. The most studied is H3T, which is encoded by two genes: H3f3a and H3f3b. Further studies produced a knock-out murine model for the H3f3b gene. The authors observed a total loss of fertility and a decreased expression of spermatogenesis-related genes, also exhibiting defective chromatin remodeling and protamination (Yuen et al., 2014). The specific histone TH2 B's experimental knockout was related to histone replacement alterations by protamines in human and murine spermatozoa (Ausio et al., 2016).

Protamine genes could also be targeted by epigenetic changes, sometimes resulting in an inefficient translation. High variability in the 5' VLR (Variable Length Repeat) region of the human PRM2 gene could lead to dysregulated protamination (Hammoud et al., 2007). The presence of SNPs (Single Nucleotide Polymorphisms) in the UTR (untranslated regions) of PRM1 or PRM2 could interfere with the splicing transcription factor, resulting in defective protamination, as related in humans (Ravel et al., 2007)

Inside the testis parenchyma, there is an endocrine regulation of spermatogenesis that could reflect on sperm DNA compaction. The interaction between follicle-stimulating hormone (FSH) and testosterone plays a regulatory role in sperm chromatin, as FSH knock-out experiments reported changes in sperm chromatin and increased DNA fragmentation in murine spermatozoa (Xing et al., 2003). Also, FSH regulates the cyclic-AMP responsive modulator element (CREM) pathway, which is responsible for regulating the transcriptional activity of protamines and other genes required for proper spermatogenesis. The alterations in the CREM gene have been related to inadequate protamination of murine spermatozoa (Blendy et al., 1996). Other regulators of protamination have also been described such as the PRM-1 RNA-binding protein (Prbp) and MSY2, both found at specific stages in murine testicular tissues (Lee et al., 1996; Yu et al., 2002). The regulation of protamination is important because, if altered, it would result in early compaction, transcriptional sequestration, and consequent failure of sperm development (Lee et al., 1995; Kleene, 2003).

### Extrinsic factors

#### Oxidative and heat stress

**Figure 2:**
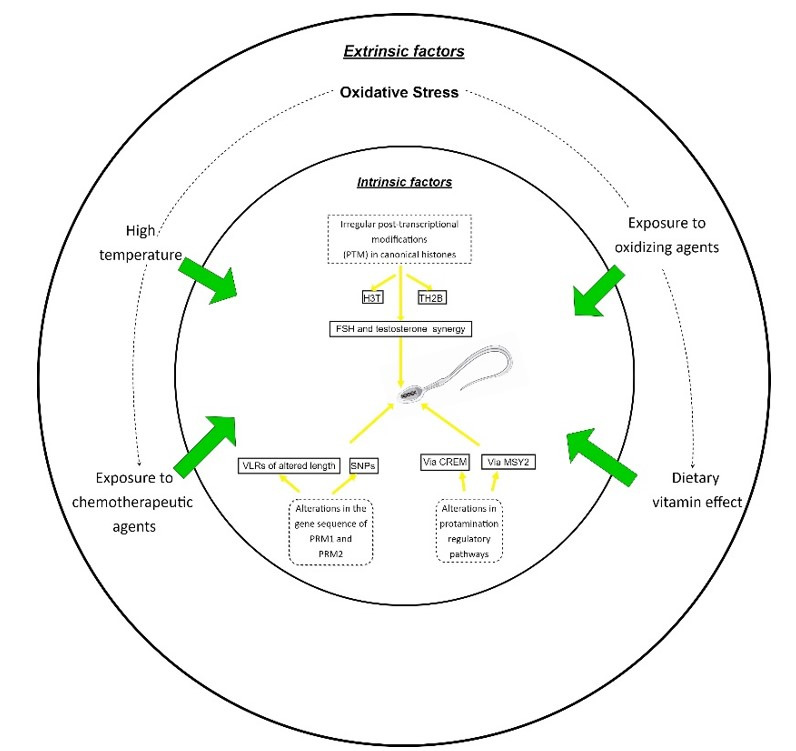
Extrinsic and intrinsic factors involved in bovine spermatozoa protamination and its effects.

A controlled environment is important as some external conditions could reflect negatively on the spermatozoa. Exposure to noxious substances or stressful conditions like heat stress could increase sperm metabolism and consequently, the production of reactive oxygen species (ROS), as suggested in Figure 2. Under harmful conditions compensatory mechanisms are triggered, such as the synthesis of heat shock proteins, oxidation of I, II, III, and IV mitochondria complexes, and changes in the cell basal metabolism, looking for homeostasis (England et al., 2004; Belhadj Slimen et al., 2016).

Nevertheless, heat stress can be present in different stages of spermatogenesis, being more deleterious in early spermatogenesis (spermatocyte and spermatid stages) irrupting transcription and transduction of various genes, yet not defined (Hikim et al., 2003; Zhu et al., 2004; Pérez-Crespo et al., 2008). Physiologically, altered spermatocytes would go under cell death after apoptotic signalization; nevertheless, as gene expression is compromised, also interferes with the identification of apoptotic-signalized, gene expression and promotes an increase of DNA breaks (Absalan et al., 2012; Kim et al., 2013).

Therefore, some heat-injured spermatozoa continue along spermatogenesis with several alterations, resulting in a dysfunctional gamete with morphological alterations, mitochondrial misfunction, and a looser chromatin (Paul et al., 2008; Rahman et al., 2011; Kim et al., 2013). Those alterations would also induce DNA breaks, especially in determinant regions where protamine-histone substitution occurs. Experimentally, a direct relationship is reported between induced testicular insulation and higher rates of DNA fragmentation, chromatin alterations, and impaired protamination (Rahman et al., 2011; Hamilton et al., 2018; Garcia-Oliveros et al., 2020). Moreover, ART techniques such as cryopreservation are also related to a reduction of antioxidative enzymes and an increase of ROS because of osmotic and oxidative stress (Bollwein and Bittner, 2018). As a result, cryopreserved semen shows sperm populations with different levels of oxidative stress (measured by TBARs) which have a positive correlation with DNA fragmentation and reduced embryo quality (Simões et al., 2013).

To compensate for the excessive production of ROS, supplementation with vitamins (known for their antioxidant function) is used as a therapeutic alternative (Greco et al., 2005). Moreover, recent studies have related the effect of some vitamins (Vitamin C and E) over regulatory mechanisms that have not been elucidated yet on spermatogenesis and protamine biosynthesis (Hamidian et al., 2020; Gao et al., 2021). Vitamin C and E supplementation would be able to improve sperm chromatin status, increasing protamine expression and decreasing the percentage of sperm with fragmented DNA (Greco et al., 2005; Saito et al., 2020).

In summary, in the sperm chromatin is happening some sequenced events: heat stress negatively modifies gene and protein expression which arises ROS production resulting in oxidative damage (Garcia-Oliveros et al., 2020). Nevertheless, those stimuli are suggested to cause marks along the sperm epigenome, reflected in increased DNA methylation, altered nucleoprotein substitution, male pronucleus delay, and final impairment in embryo development (Rahman et al., 2014; Kiefer et al., 2021). The resulting epigenetic profile varies among individuals, because of the degree of susceptibility to external or noxious stimuli.

### Protamination check-up

Protamination status is not checked in routine semen evaluation, but experimental approaches consider direct or indirect evaluation. Direct evaluation has been validated already in humans and murine through absolute quantification of protamines by qPCR-RT, as the relative expression is not appropriate due to the reduced transcription activity in mature spermatozoa (Ni et al., 2016). It is a useful biomarker as altered ratios are related to infertility (Aoki et al., 2005, 2006; Amor et al., 2018). Nevertheless, the relationship between protamination and fertility in bulls is a new topic, being at first described by relative values and recently through absolute quantifications (Hamilton et al., 2019). As protamines are basic proteins, they also can be measured by a modified western blot because protamines are negative-charged proteins, being been already validated for bulls (Hamilton et al., 2021).

On the other hand, protamination status can be verified indirectly through chromatin integrity tests, such as toluidine blue, acridine orange, sperm chromatin structure assay, chromomycin A3 test, sperm chromatin dispersion test, COMET assay, and TUNEL assay (Souza et al., 2018). Among those tests, chromomycin A3 and acridine orange users extended and validated for human and murine research; also, these dyes give important information about chromatin status (protamine deficiencies and sperm susceptibility to fragmentation) as they help to estimate male fertility (Ni et al., 2016). Therefore, predicting bull fertility is still difficult due to adaptative traits among breeds, *male* factors, and the poor understanding of sperm trait interactions. Despite this, chromomycin A3 is described as a useful test to measure chromatin packaging and is correlated to bull fertility (Llavanera et al., 2021).

## Conclusions

Research, based on human and murine spermatozoa, describes the importance of the composition and integrity of sperm chromatin as it may not interfere with fertilization but with embryo development. Nevertheless, the dynamics in bull sperm chromatin are still poorly understood. What is clear, is that chromatin maturation depends on a preserved protamination (mainly to PRM1). Therefore, this review described the mechanisms that may impair protamination and consequently chromatin maturation. So far, it is acknowledged that external stimulus (ie: heat stress) and dysregulation of intrinsic factors may alter spermatogenesis, and gene expression and finally impair protamination. Also, the current methods (direct and indirect) of protamine evaluation are important but it is suggested that some functional studies are needed to achieve a better understanding of protamine dynamics in bulls.
